# Application of digital infrared thermography for carpal tunnel syndrome evaluation

**DOI:** 10.1038/s41598-021-01381-5

**Published:** 2021-11-09

**Authors:** Dougho Park, Byung Hee Kim, Sang-Eok Lee, Dong Young Kim, Yoon Sik Eom, Jae Man Cho, Joong Won Yang, Mansu Kim, Heum Dai Kwon, Jang Woo Lee

**Affiliations:** 1 Department of Rehabilitation Medicine, Pohang Stroke and Spine Hospital, Pohang, Republic of Korea; 2 Department of Orthopedic Surgery, Pohang Stroke and Spine Hospital, Pohang, Republic of Korea; 3 Department of Neurosurgery, Pohang Stroke and Spine Hospital, Pohang, Republic of Korea; 4 Department of Physical Medicine and Rehabilitation, National Health Insurance Service Ilsan Hospital, Goyang, Republic of Korea

**Keywords:** Peripheral neuropathies, Chronic pain

## Abstract

We investigated the thermographic findings of carpal tunnel syndrome (CTS). We enrolled 304 hands with electrodiagnostically identified CTS and 88 control hands. CTS hands were assigned to duration groups (D1, < 3 months; D2, 3‒6 months; D3, 6‒12 months; D4, ≥ 12 months) and severity groups (S1, very mild; S2, mild; S3, moderate; S4, severe). The temperature difference between the median and ulnar nerve territories (ΔM-U territories) decreased as CTS duration and severity increased. Significant differences in ΔM-U territories between the D1 and D3, D1 and D4, D2 and D4, and S1 and S4 groups (*P* = 0.003, 0.001, 0.001, and < 0.001, respectively) were observed. Thermal anisometry increased as CTS duration and severity increased. Significant differences in thermal anisometry between the D1 and D4 as well as the D2 and D4 groups (*P* = 0.005 and 0.04, respectively) were noted. Thermal anisometry was higher in the S4 group than in the S1, S2, and S3 groups (*P* = 0.009, < 0.001, and 0.003, respectively). As CTS progresses, skin temperature tends to decrease and thermal variation tends to increase in the median nerve-innervated area. Thermographic findings reflect the physiological changes of the entrapped median nerve.

## Introduction

Carpal tunnel syndrome (CTS), which is the most common entrapment neuropathy, is caused by compression of the median nerve in the wrist^1, 2^. Neurogenic pain, paresthesia, and numbness in the median nerve-innervated area are typical CTS symptoms. With chronic CTS or severe neural compression, thenar muscle weakness and/or atrophy may occur.

One of the reliable and objective methods for assessing CTS is electrodiagnosis, which reflects the neurophysiological functional status and is useful for grading severity^3, 4^. Ultrasonography (US) is also widely used because it can quantitatively evaluate swelling of the median nerve and carpal tunnel outlet, and it allows guided administration of the injection through real-time visualization^5–7^. Electrodiagnosis, however, can mainly detect pathologies in thick-myelinated fibers; its sensitivity for detecting injuries of thin-unmyelinated fiber is relatively low^8^. Although US allows direct visualization of the morphology of the median nerve and carpal tunnel, its ability to assess the neurophysiological state is limited^9^.

Digital infrared thermographic imaging (DITI) measures body heat emission of the skin surface and visually expresses that heat in the digitalized form^10^, thus reflecting the physiological changes in the target body part^11^. Hence, it is used to evaluate various diseases, such as breast cancer, diabetic microvascular disease, complex regional pain syndrome, arthritic pain, and myofascial pain syndrome^12–15^. DITI has also been used to evaluate peripheral nerve injury. In particular, autonomic dysfunction accompanying peripheral neuropathy, which is an important mechanism that causes changes in skin temperature by affecting vasomotor activity, is reflected by DITI^16, 17^.

Compared to other methods, such as electrodiagnosis and US, DITI has the distinctive ability to evaluate the status of the autonomic nervous system, which consists of thin-unmyelinated fibers^18^. Several studies have attempted to investigate the clinical usefulness of DITI for CTS; however, currently, DITI has not shown consistent clinical value for CTS^19, 20^. During the early stage of CTS, thin-unmyelinated fibers are damaged and thick-myelinated fibers are injured as the disease progresses^8, 9, 21^. We thought that these inconsistent results were based on the lack of consideration of temporal neurophysiological changes during CTS.

During this study, we investigated the DITI results according to symptom duration and disease severity in hands with CTS. Additionally, the relationships between DITI values and other parameters, including US findings and self-reported pain scores, were evaluated to verify the potential of DITI as a tool for evaluating CTS.

## Methods

### Subject inclusion and ethical approval statements

**Figure 1:**
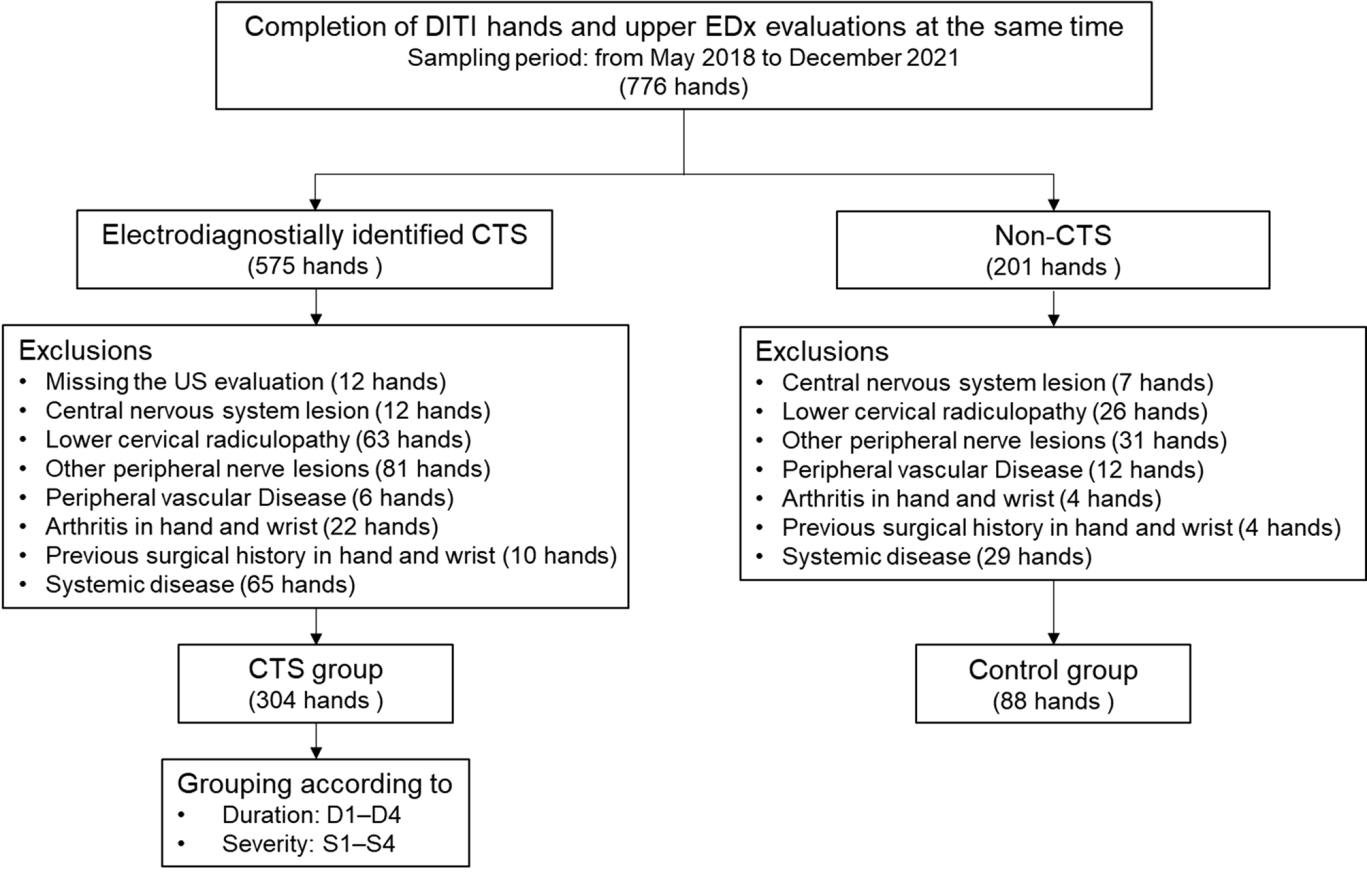
Flow chart of the patient selection process. *DITI* digital infrared thermography, *EDx* electrodiagnosis, *CTS* carpal tunnel syndrome, *US* ultrasonography, *D* symptom duration group, *S* severity group.

The process of subject inclusion for this study is shown in Fig. 1. All clinical data used for this retrospective study were obtained from a single hospital from May 2018 to December 2020. From the dataset, we extracted patients who were diagnosed with CTS using electrodiagnosis and who were simultaneously evaluated using DITI of the affected hand or hands. During the first visit, the degree of pain was recorded using a numeric rating scale and related clinical symptoms were evaluated. We also checked whether decompression surgery was performed for the hand with CTS within 6 months after the electrodiagnosis. We excluded hands with the following characteristics: central nervous system lesion affecting the hands; concomitant lower cervical radiculopathy or other peripheral nerve lesions; peripheral vascular disease; arthritis in the hand and wrist; previous surgery of the wrist or hand; systemic diseases, such as tumors, thyroid diseases, fibromyalgia, and uncontrolled diabetes mellitus (glycosylated hemoglobin > 9%); and missing US evaluations. After exclusion, the included CTS hands were classified into each subgroup according to the period from symptom onset to the time of diagnostic examinations and electrodiagnostically defined severity. Subgroups according to the symptom duration were defined as follows: D1, < 3 months; D2, 3 to < 6 months; D3, 6 to < 12 months; and D4, ≥ 12 months.

Subjects in the control group also underwent DITI of the hands and electrodiagnosis evaluations during the same sampling period. The control group was subjected to the same evaluation protocols for DITI and electrodiagnosis as those for the patients with CTS. Hands that did not show CTS according to the electrodiagnosis results were first extracted from the dataset. Then, exclusion criteria for final inclusion were equally applied to the controls.

This study was approved by the Institutional Review Board of Pohang Stroke and Spine Hospital (approval no.: PSSH0475-202101-HR-002-01) and retrospectively registered at cris.nih.go.kr (identifier: KCT0005831; January 20, 2021). Because of the retrospective study design, the Institutional Review Board of Pohang Stroke and Spine Hospital allowed the omission of informed consent. This study was performed in compliance with the Declaration of Helsinki and the International Conference on Harmonization-Good Clinical Practice Guideline. The dataset supporting the findings of this study is available in the online supplementary content.

### Electrodiagnosis and carpal tunnel syndrome severity

To obtain compound motor nerve action potential from the abductor pollicis brevis (APB) muscle, the median nerve was stimulated at a distance of 8 cm proximal to the APB. Normal values were onset latency ≤ 4.0 ms and amplitude ≥ 5 mV. To induce sensory nerve action potential, a recording electrode was placed on the second digit, and stimulation was performed at the wrist at 14 cm proximal to the recording electrode. Normal values were onset latency ≤ 3.5 ms and amplitude ≥ 20 µV. For transcarpal latency acquisition, an additional test was performed at 7 cm proximal to the sensory nerve action potential recording site at the palm. A transcarpal latency value of ≥ 1.7 ms diagnostically confirmed CTS. For patients with obvious CTS-related symptoms but with transcarpal latency values within the normal range (≤ 1.5 ms to < 1.7 ms), a lumbrical interossei comparison study and ring finger study were additionally conducted as sensitivity tests. During the lumbrical interossei comparison study, an active recording electrode was attached to the midpoint of the third metacarpal bone and a reference recording electrode was attached to the second proximal interphalangeal joint to stimulate the median nerve and ulnar nerve, respectively, at the wrist. CTS was diagnosed when the difference in onset latency between the two stimulations was > 0.4 ms. During the ring finger study, data were recorded at the fourth digit and the median and ulnar nerves were stimulated at 14 cm proximal to the recording electrode at the wrist. CTS was diagnosed when the difference in onset latency between the two stimulations was ≥ 0.6 ms. Abnormal spontaneous electrical activities and regeneration potentials were evaluated using needle electromyography of the APB muscle^22^.

Electrodiagnosis was conducted using Sierra® wave (Cadwell, Kennewick, WA, USA). We maintained the laboratory temperature at 23–25 °C. All patients were placed in the supine position during the tests. A nerve conduction study of the ulnar, radial, and median nerves and electromyography of the muscles corresponding to each cervical root level were performed to exclude other differential diagnoses. Electrodiagnosis was conducted after the DITI tests.

We grouped the electrodiagnostic severity of CTS using a partial modification of the Stevens classification^23^. The very mild (S1) group included cases with only prolonged transcarpal latency or abnormal sensitivity test findings. The mild (S2) group included cases with abnormal transcarpal latency or sensitivity test findings, with prolonged-onset latency or decreased amplitude of the sensory nerve action potential, and without abnormal findings of the compound motor nerve action potential of the median nerve. The moderate (S3) group included cases with abnormal transcarpal latency or sensitivity test findings, with abnormalities of the compound motor nerve action potential, and without abnormal findings of needle electromyography of the APB muscle. The severe (S4) group included those with abnormal transcarpal latency or sensitivity test findings and abnormal findings of needle electromyography of the APB muscle^24^.

### US evaluation

US evaluation was conducted for the patient group using iU22 (Philips, Bothell, WA, USA) with a linear (12–5 MHz) probe. Patients were asked to assume an upright sitting position and instructed to bend their elbows at 90° and fully supinate their forearms. The pisiform and scaphoid were identified at the level just proximal to the carpal tunnel, and transverse images were acquired to measure the cross-sectional area (CSA) of the median nerve^24, 25^.

### Evaluation protocol and DITI interpretation

The IRIS-8000® (Medicore, Seoul, Korea) was used to obtain the thermographic image. DITI was performed before electrodiagnosis with the following conditions: the indoor temperature was maintained at 23–25 °C; the subject was dressed in loose clothing and allowed to acclimatize to the room temperature for 15‒20 min; the distance from the body to the thermographic camera was 1.5 m; lotion or ointment was not applied before the test; all metal accessories, splints, and topical patches were removed; vigorous exercise and physical therapy within 4 h of the test were avoided; and alcohol and caffeine consumption and smoking were prohibited within 12 h before the examination.

**Figure 2:**
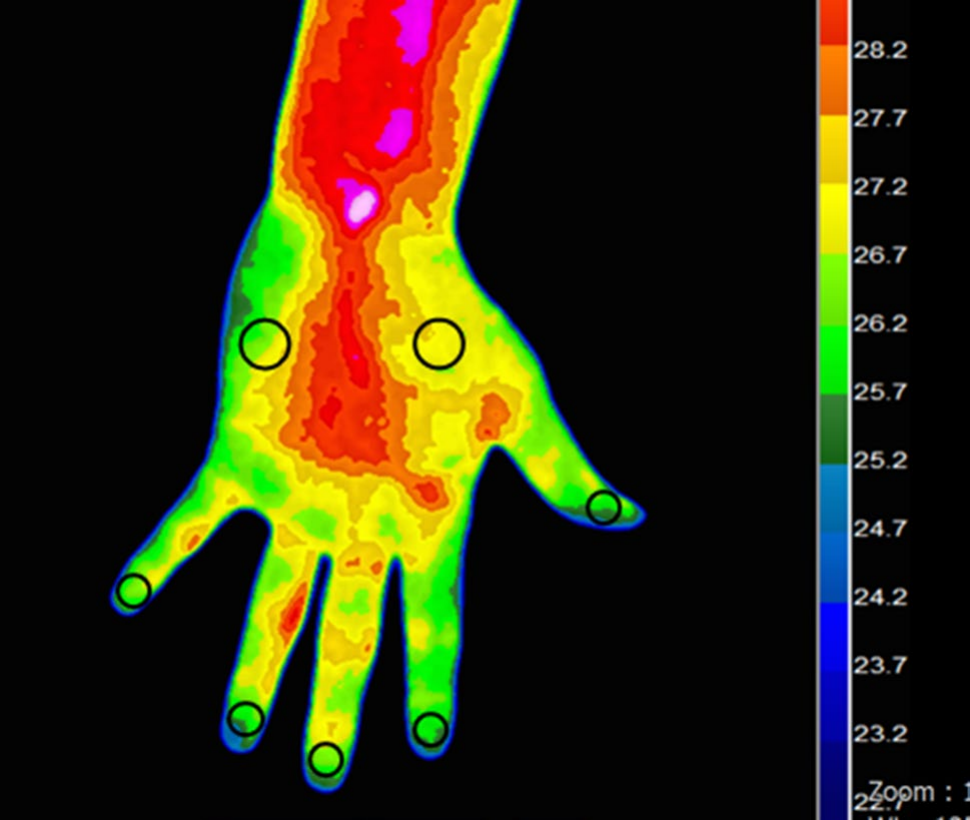
Digital infrared thermographic imaging and regions of interest of the palm area. The mean temperature is measured at the center of the finger pulp, thenar eminence, and hypothenar eminence (black circles).

Because previous studies reported that median-innervated somatosensory and vasomotor territories are similar in the palm area^26, 27^, we designated six regions of interest of the thermal image of the palm side. Skin temperature was measured at the center of the finger pulp of the first, second, third, and fifth digits based on an area of 400 data points. Additionally, measurements were obtained from the thenar eminence and hypothenar eminence based on an area of 800 data points (Fig. 2). The mean value of all data points in the region of interest was calculated. The final result is displayed in degrees (Celsius) by converting the validated red–green–blue data to temperature. For data interpretation and quantitative analyses, we defined the following indicators and calculated the values by substituting the average body temperature measured within each region of interest^9^:

$${\text{Temperature }}\;{\text{difference}}\;{\text{ between }}\;{\text{the }}\;{\text{median }}\;{\text{and}}\;{\text{ ulnar}}\;{\text{ nerve }}\;{\text{digits }}\left( {\Delta {\text{M}} - {\text{U}}\;{\text{ digits}}} \right) = \, \left( {{\text{digit }}1 \, + {\text{ digit }}2 \, + {\text{ digit }}3 \, {-} \, 3 \times {\text{digit }}5} \right)/3,$$

$${\text{Temperature}}\;{\text{difference}}\;{\text{between}}\;{\text{the}}\;{\text{thenar}}\;{\text{and}}\;{\text{hypothenar}}\;{\text{areas }}(\Delta {\text{thenar - hypothenar}}) \, = {\text{ thenar}} - {\text{hypothenar,}}$$

$${\text{Temperature }}\;{\text{difference}}\;{\text{ between }}\;{\text{the}}\;{\text{ median }}\;{\text{and}}\;{\text{ ulnar}}\;{\text{ nerve }}\;{\text{territories }}(\Delta {\text{M}} - {\text{U }}\;{\text{territories}}) \, = \Delta {\text{M}} - {\text{U }}\;{\text{digits}} + \Delta {\text{thenar}} - {\text{hypothenar,}}$$

$${\text{Median }}\;{\text{nerve - innervated}}\;{\text{ digits }}\;{\text{anisometry }} = \, \left| {{\text{digit }}1 - {\text{digit }}2} \right| \, + \, \left| {{\text{digit }}1 - {\text{digit }}3} \right| \, + \, \left| {{\text{digit }}2 - {\text{digit }}3} \right|.$$

### Statistical analysis

Continuous variables are expressed as mean ± standard deviation. Categorical variables are expressed as frequency and proportion. An independent *t*-test was used to compare the age and DITI values between the patient and control groups. The chi-square test was performed to compare the sex and investigated side of the patients with those of the controls. Cramer’s V coefficient was used to examine the association between the duration and severity groups. A one-way analysis of variance with Bonferroni correction was used to analyze DITI indicators according to symptom duration and electrodiagnostic severity. The Pearson correlation coefficient was used to analyze the correlations among the DITI indicators, CSA, and numeric pain scale rating. All statistical analyses were conducted using SPSS 22.0 (IBM, Armonk, NY, USA).

## Results

### General characteristics of the subjects

A total of 304 CTS hands of 210 patients and a total of 88 normal hands of 80 healthy control individuals were finally included in this study. The mean age of the CTS patients was 57.2 ± 10.2 years, 24.3% were male, and the left and right hands were evenly distributed. The average age of the control group was 55.0 ± 10.9 years, 26.3% were male, and there were slightly more right hands (55.7%) than left hands. The patient group showed a significantly higher body mass index and prevalence of hypertension. The average CSA of the investigated hands was 15.7 ± 4.4 mm^2^. The average numeric pain scale rating of the CTS group was 4.8 ± 1.6. No statistically significant differences in age, sex, and investigated side were found between the two groups. Among hands with CTS, 68 (22.4%) underwent decompression surgery within 6 months after the initial electrodiagnosis (Table 1).

**Table 1:** Characteristics of the subjects.

	Patients	Healthy controls	*P* value
Subjects, n	210	80	NA
Hands, n	304	88	NA
NRS^a^	4.8 ± 1.6	NA	NA
CSA, mm^2a^	15.7 ± 4.4	NA	NA
Decompression, n (%)	68 (22.4)	NA	NA
Investigated hand, right, n (%)	152 (50.0)	49 (55.7)	0.35
Age, years^a^	57.2 ± 10.2	55.0 ± 10.9	0.12
Male, n (%)	51 (24.3)	21 (26.3)	0.73
Body mass index, kg/m^2^	25.11 ± 3.36	24.11 ± 4.06	0.04
Hypertension, n (%)	58 (27.6)	12 (15.0)	0.03
Well-controlled DM, n (%)	25 (11.9)	6 (7.5)	0.28
Dyslipidemia, n (%)	21 (10.0)	8 (10.0)	> 0.99
Smoking, n (%)	24 (11.4)	6 (7.5)	0.33

*NA* not applicable, *NRS* numerical rating scale of pain, *CSA* cross-sectional area of the median nerve, *DM* diabetes mellitus.

^a^All continuous values were expressed as mean ± standard deviation.

Distributions according to the CTS duration and severity are shown in Table 2. Based on the duration, there were 91 hands in the D1 group, 59 hands in the D2 group, 57 hands in the D3 group, and 97 hands in the D4 group. Based on the severity, there were 73 hands in the S1 group, 60 hands in the S2 group, 83 hands in the S3 group, and 88 hands in the S4 group. A longer disease duration was associated with increased severity (Cramer’s V = 0.35; *P* < 0.001).

**Table 2:** Number of involved hands according to disease severity and symptom duration.

Duration groups	Severity groups				
	Very mild (S1)	Mild (S2)	Moderate (S3)	Severe (S4)	Total
D1 (< 3 months)	40	27	19	5	91
D2 (3–6 months)	15	13	27	4	59
D3 (6–12 months)	9	12	18	18	57
D4 (≥ 12 months)	9	8	19	61	97
Total	73	60	83	88	304

### DITI findings

**Figure 3:**
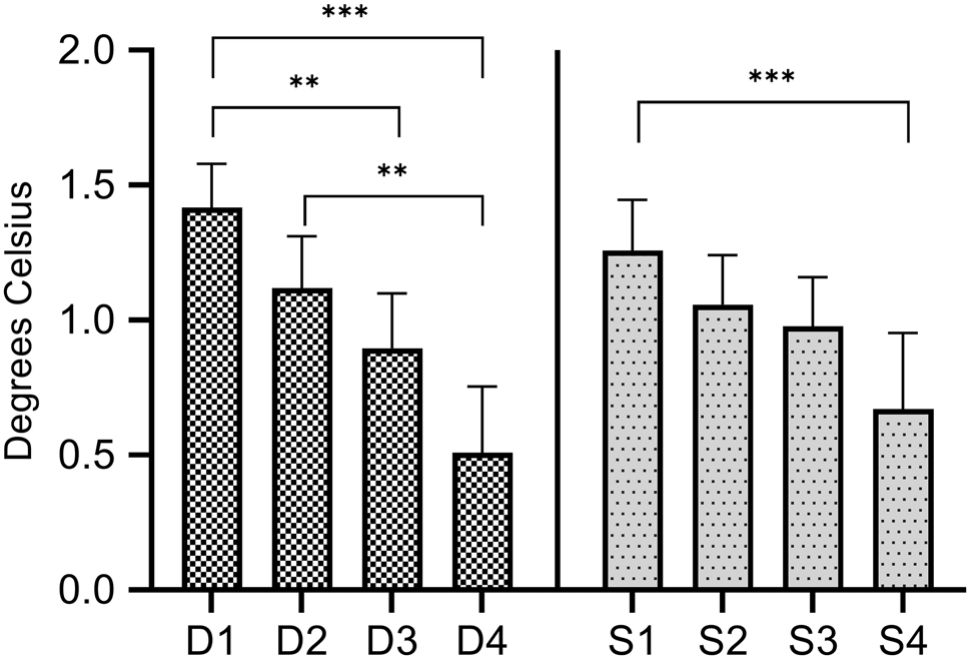
Comparisons of temperature differences between the median and ulnar nerve territories (ΔM-U territories) according to the symptom duration and disease severity groups. Significant differences in ΔM-U territories between the D1 and D3, D1 and D4, D2 and D4, and S1 and S4 groups are observed. This graph is drawn using GraphPad Prism version 9.1.2 for Windows (GraphPad Software, San Diego, California USA, www.graphpad.com). *D* duration group, *S* severity group. *P < 0.05. **P < 0.01. ***P < 0.001.

The ΔM-U territories was significantly greater in all patient groups compared to the control group. Greater ΔM-U territories was associated with shorter duration and lower severity (Table 3). The ΔM-U territories was significantly different between the D1 and D3 group, D1 and D4 group, and D2 and D4 group (*P* = 0.003, < 0.001, and 0.001, respectively). Only the S1 and S4 groups had significant differences in ΔM-U territories among the severity groups (*P* < 0.001) (Fig. 3).

**Table 3:** Values of each index according to the symptom duration and disease severity groups.

Group	ΔM-U digits (℃)^a^	*P* value^b^	Δthenar-hypothenar (℃)^a^	*P* value^b^	ΔM-U territories (℃)^a^	*P* value^b^	Anisometry (℃)^a^	*P* value^b^
**Duration groups**								
D1 (n = 91)	0.91 ± 0.56	< 0.001	0.51 ± 0.50	< 0.001	1.41 ± 0.78	< 0.001	1.29 ± 0.84	0.01
D2 (n = 59)	0.68 ± 0.57	< 0.001	0.44 ± 0.42	0.006	1.12 ± 0.74	< 0.001	1.32 ± 1.14	0.03
D3 (n = 57)	0.59 ± 0.49	< 0.001	0.31 ± 0.43	0.40	0.90 ± 0.77	< 0.001	1.37 ± 0.72	0.01
D4 (n = 97)	0.39 ± 0.68	0.008	0.12 ± 0.79	> 0.99	0.51 ± 1.22	0.03	1.79 ± 1.35	< 0.001
**Severity groups**								
S1 (n = 73)	0.86 ± 0.58	< 0.001	0.39 ± 0.49	0.02	1.26 ± 0.81	< 0.001	1.37 ± 0.96	0.004
S2 (n = 60)	0.61 ± 0.47	< 0.001	0.45 ± 0.42	0.005	1.06 ± 0.71	< 0.001	1.17 ± 0.93	0.300
S3 (n = 83)	0.63 ± 0.59	< 0.001	0.35 ± 0.48	0.07	0.98 ± 0.83	< 0.001	1.34 ± 0.79	0.005
S4 (n = 88)	0.48 ± 0.73	< 0.001	0.19 ± 0.84	> 0.99	0.67 ± 1.33	0.001	1.88 ± 1.38	< 0.001
**Healthy controls (n = 88)**	0.11 ± 0.40		0.12 ± 0.37		0.23 ± 0.62		0.82 ± 0.55	

*D* symptom duration group, *S* severity group, *ΔM-U digits* temperature difference between the median and ulnar nerve digits, *Δthenar-hypothenar* temperature difference between the thenar and hypothenar areas, *ΔM-U territories* temperature difference between the median and ulnar nerve territories, *Anisometry* median nerve-innervated digits anisometry.

^a^All continuous values were expressed as mean ± standard deviation.

^b^Independent *t*-test between each patient group and the control group.

**Figure 4:**
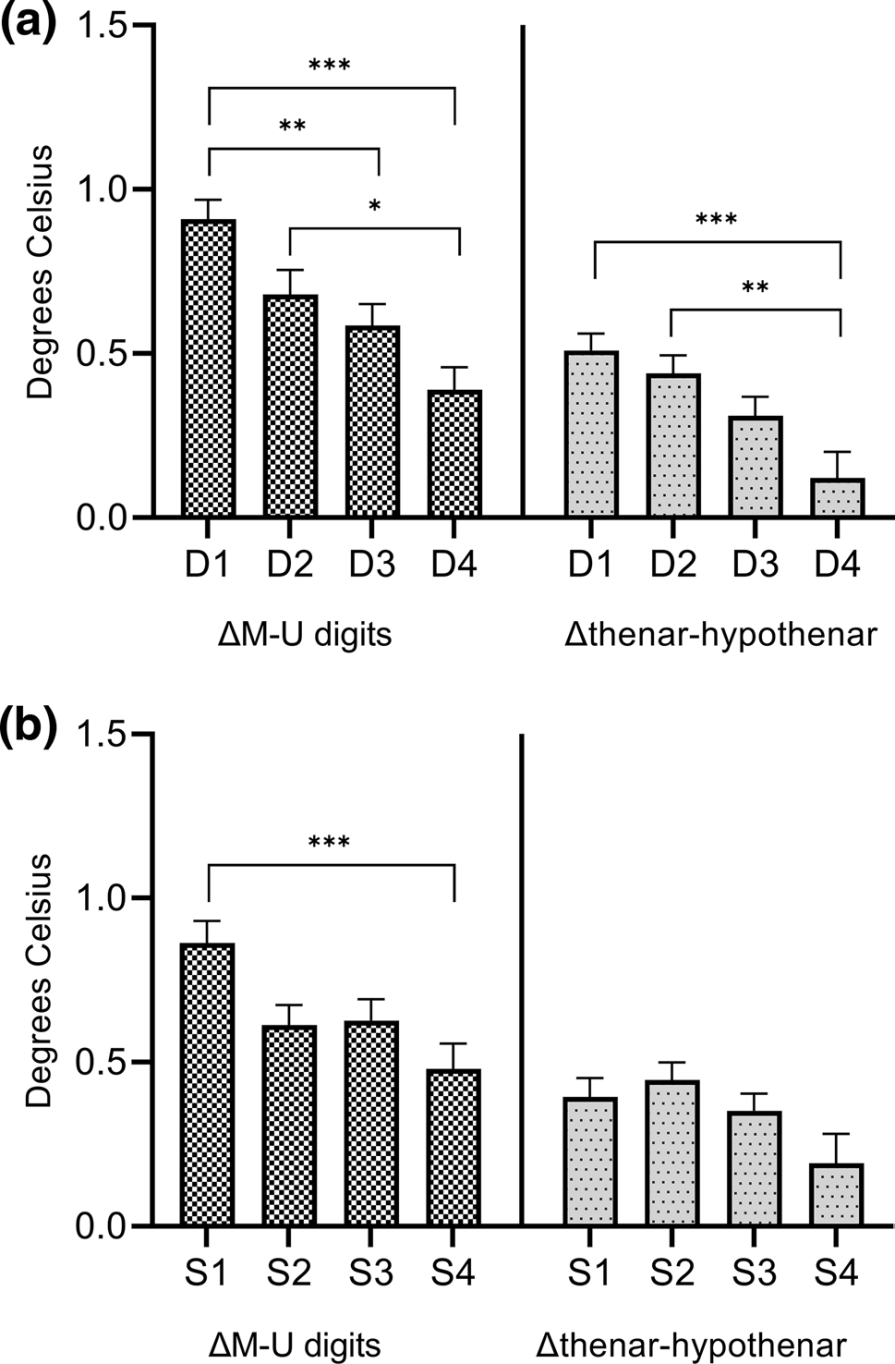
Comparisons of the temperature differences between the median and ulnar nerve digits (ΔM-U digits) and temperature differences between the thenar and hypothenar areas (Δthenar-hypothenar) according to duration (**a**) and severity (**b**). Significant differences in ΔM-U digits between the D1 and D3, D1 and D4, D2 and D4, and S1 and S4 groups are observed. Significant differences in Δthenar-hypothenar between the D1 and D4 groups and between the D2 and D4 groups are also observed. This graph is drawn using GraphPad Prism version 9.1.2 for Windows (GraphPad Software, San Diego, California USA, www.graphpad.com). D, duration group; S, severity group. *P < 0.05. **P < 0.01. ***P < 0.001.

Moreover, ΔM-U digits was significantly larger in all patient groups compared to the control group. No significant difference in Δthenar-hypothenar between the D3, D4, S3, and S4 groups and the control group was observed. Similar to ΔM-U territories values, ΔM-U digits and Δthenar-hypothenar decreased as the duration increased. A comparison of the duration groups showed that ΔM-U digits was significantly different between the D1 and D3 group, D1 and D4 group, and D2 and D4 group (*P* = 0.006, 0.001, and 0.02, respectively), and that Δthenar-hypothenar was significantly different between the D1 and D4 groups and between the D2 and D4 groups (*P* < 0.001 and 0.004, respectively) (Table 3 and Fig. 4a). Furthermore, based on the changes in ΔM-U digits and Δthenar-hypothenar based on severity, the S2 group had lower ΔM-U digits than the S3 group and higher Δthenar-hypothenar than the S1 group. ΔM-U digits was significantly different between the S1 and S4 groups (*P* < 0.001), whereas Δthenar-hypothenar was not statistically significantly different between all severity groups (Table 3 and Fig. 4b).

**Figure 5:**
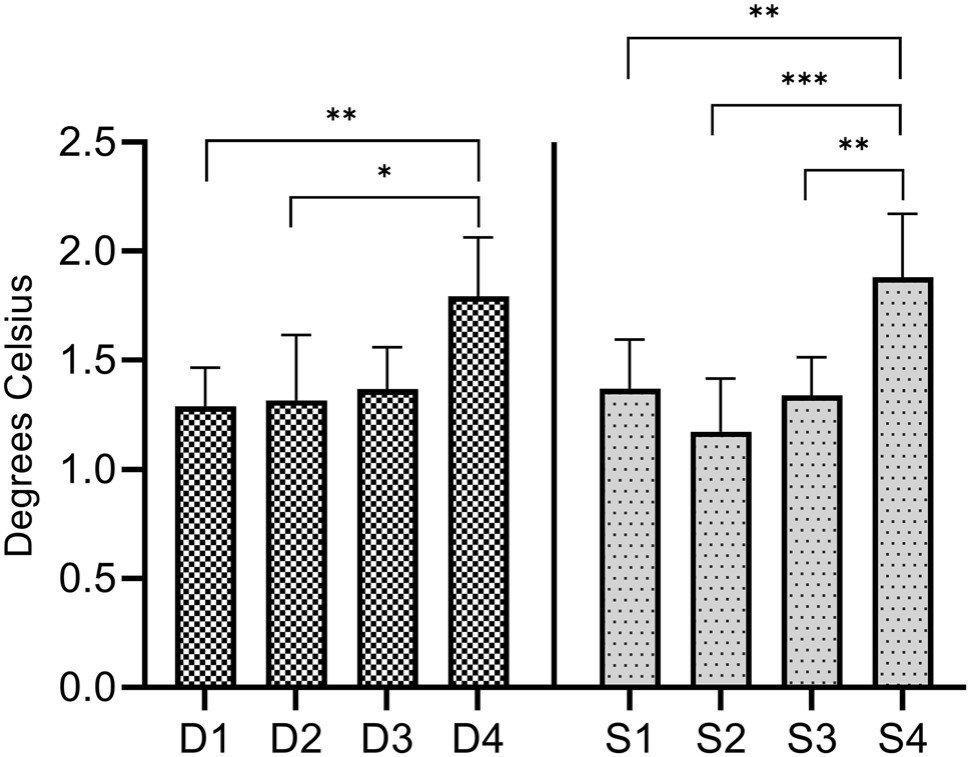
Median nerve-innervated digits anisometry according to duration and severity. Significant differences in the median nerve-innervated digits anisometry between the D1 and D4 groups and between the D2 and D4 groups are observed. The S4 group also has significantly higher median nerve-innervated digits anisometry than the S1, S2, and S3 groups. This graph is drawn using GraphPad Prism version 9.1.2 for Windows (GraphPad Software, San Diego, California USA, www.graphpad.com). D, duration group; S, severity group. *P < 0.05. **P < 0.01. ***P < 0.001.

The median nerve-innervated digits anisometry increased as the duration increased and was significantly higher in all duration groups than in the control group. Among the severity groups, the S2 group had the lowest median nerve-innervated digits anisometry, and no significant difference between the S2 group and control group was observed (*P* = 0.30). Median nerve-innervated digits anisometry was significantly higher in the S1, S3, and S4 groups than in the control group. For the duration groups, median nerve-innervated digits anisometry was significantly different between the D1 and D4 groups and between the D2 and D4 groups (*P* = 0.005 and 0.04, respectively), and in the severity groups. The median nerve-innervated digits anisometry was significantly higher in the S4 group than in the S1, S2, and S3 groups (*P* = 0.009, < 0.001, and 0.003, respectively) (Table 3 and Fig. 5).

The hands with CTS undergoing decompression surgery showed significantly lower Δthenar-hypothenar and ΔM-U territories values (*P* = 0.007 and 0.008, respectively). Contrarily, the median nerve-innervated digits anisometry was significantly higher in the decompression group (*P* = 0.005) (Table 4).

**Table 4:** Values of each index according to the surgical decision.

	Decompression (n = 68)	None (n = 236)	*P* value
ΔM-U digits (℃)^a^	0.53 ± 0.65	0.67 ± 0.62	0.11
Δthenar-hypothenar (℃)^a^	0.16 ± 0.83	0.38 ± 0.52	0.007
ΔM-U territories (℃)^a^	0.69 ± 1.26	1.05 ± 0.69	0.008
Anisometry^a^	1.80 ± 1.22	1.38 ± 1.03	0.005

^a^All continuous values were expressed as mean ± standard deviation.

The DITI values of each region of interest according to the CTS duration and severity are summarized in Supplementary Table S1. The results of post hoc analyses of subgroups are summarized in Supplementary Table S2.

### Correlations between examined parameters and the pain score

The ΔM-U territories showed weak negative correlations with CSA (*r* =  − 0.14; *P* = 0.02) and the numeric pain scale rating (*r* =  − 0.14; *P* = 0.01). No significant correlation between ΔM-U territories and median nerve-innervated digits anisometry was noted (*r* = 0.03; *P* = 0.56). Moreover, the numeric pain scale rating showed a weak positive correlation with CSA (*r* = 0.29; *P* < 0.001) (Table 5). The CSA and numeric pain scale rating according to the CTS duration and severity subgroups are summarized in Supplementary Table S3.

**Table 5:** Correlation coefficients (*P* values) between the examined parameters and pain score.

	ΔM-U territories	Anisometry	CSA	NRS
ΔM-U territories	1	0.03 (0.56)	− 0.14 (0.02)	− 0.14 (0.01)
Anisometry	0.03 (0.56)	1	0.09 (0.11)	0.06 (0.33)
CSA	− 0.14 (0.02)	0.09 (0.11)	1	0.29 (< 0.001)
NRS	− 0.14 (0.01)	0.06 (0.33)	0.29 (< 0.001)	1

*ΔM-U territories* temperature difference between the median and ulnar nerve digits, *Anisometry* median nerve-innervated digits anisometry, *CSA* cross-sectional area of the median nerve, *NRS* numerical rating scale of pain.

## Discussion

Our study revealed that the CTS duration and its electrodiagnostic severity are important factors when interpreting DITI, suggesting that this may be associated with progressive entrapment neuropathy^28, 29^. The thermal difference between the median nerve-innervated and ulnar nerve-innervated areas was higher during the early phase of CTS and then decreased. Conversely, the median nerve-innervated digits anisometry tended to be increased as CTS progressed. These findings of thermal difference pattern were also consistent when comparing the groups with and without decompression surgery. By analyzing DITI results based on the disease duration and severity, we found patterns of changing DITI findings in CTS hands. Furthermore, we suggest that these results support the role of DITI as an evaluation tool for CTS. Moreover, to our knowledge, among CTS studies on DITI, this study was conducted with the largest number of hands.

The most significant finding of our research was that the ΔM-U territories was evident during the early phase of the disease but decreased as the duration increased. This is because a relative temperature increase occurs in the median innervated palm area at the beginning of the neural insult^26^. Similarly, it has been observed during an animal study that showed a slightly higher or similar temperature of the limb with a constrictive injury of the sciatic nerve lasting 28 days after injury compared to the unaffected limb^30^. However, the more severe complete injury resulted in pronounced hypothermia of the affected limb. It is believed that the temperature change at the area affected with CTS is primarily caused by vasomotor activity regulation^17^. During the early and mild phases, thin-unmyelinated fibers of the median nerve, which mainly consist of sympathetic nerve fibers, are more vulnerable to compression^31, 32^. Damage to the sympathetic nerve fibers decreases vasoconstriction in the innervated area, thereby increasing temperature in the affected area^33, 34^. The activation of antidromic unmyelinated C-fibers is also an important mechanism causing temperature changes with entrapment neuropathies^35^. Neural pathology induces pain; subsequently, vasodilators are secreted from the terminal antidromic C-fibers, resulting in an increase in local temperature^8, 9^. However, as CTS progresses, reactive vasoconstriction occurs, which is associated with hypersensitivity to catecholamines and results in a decreased temperature for several months^36^. Additionally, in chronic denervated muscles, perfusion is reduced as the capillary density in the muscle decreases^37^. A previous study also found that the low energy consumption of the myocytes leads to reduced heat emission^38^.

We inferred that the change in ΔM-U territories was more related to the change in ΔM-U digits than to the change in Δthenar-hypothenar. Compared to ΔM-U digits, Δthenar-hypothenar showed less significant differences between patients and controls. The digital cutaneous branch, which innervates the fingers and certain areas of the palm, passes through the carpal tunnel, whereas the palmar cutaneous branch, which mainly innervates the thenar area, branches from the median nerve proximal to the carpal tunnel^39^. Because the sensory innervation of the thenar area is not affected by the compression of the carpal tunnel, the Δthenar-hypothenar seems relatively small compared to the ΔM-U digits. As CTS progressed, the hypothermic effect of the thenar area was prominent. We inferred that this was mainly attributable to atrophied thenar muscles.

Contrary to ΔM-U territories, median nerve-innervated digits anisometry was more apparent as the disease progressed. This suggests that as the disease progresses, various mechanisms caused by damage in thin-unmyelinated nerve fibers, such as sympathetic dysfunction, reactive vasoconstriction, and antidromic sensory fiber activation, appear to activate simultaneously, and that thermal variations may increase even within the same nerve-innervated area. It has been reported that thermal anisometry is an early sign associated with vasoregulation^40^. Our findings were consistent with those of previous studies because thermal anisometry occurs even during the early stages of disease and may become worse as disease progresses. Moreover, some studies have reported that median nerve-innervated digits anisometry decreased after carpal tunnel release surgery, resulting in an improvement in vasoregulation by thin-unmyelinated fibers^41, 42^.

We used the symptom durations of 3 months, 6 months, and 12 months because previous studies have shown that reactive vasoconstriction occurs at 5 to 8 months^9, 43^. Symptom duration groups experienced more significant changes associated with ΔM-U territories and thermal anisometry compared to the electrodiagnostic severity groups. With compressive neuropathy, nerve fibers associated with autonomic function could be involved at the onset, which could progress and damage thick-myelinated fibers as the disease advances^34, 44^. Therefore, DITI findings could be largely dependent on the prevalent period. However, electrodiagnosis is not able to demonstrate cutaneous thermal changes because it mainly reflects the status of thick-myelinated nerve fibers^8^. Although electrodiagnosis is widely utilized to confirm or rule out CTS, its low sensitivity for injuries of thin-unmyelinated fibers affected during the early phase of the disease is a limitation^45^. To overcome this, studies aiming to confirm autonomic dysfunction with CTS using the sympathetic skin response have been conducted; however, the results are debatable^31, 46^. Attempts to identify unmyelinated fiber dysfunction with CTS using indicators, such as the thresholds of cold and warm sensations and pin-prick sensations, have also been made^47^, and supporting results have been found^45, 48^.

Efforts have been made to assess autonomic dysfunction with CTS through the use of DITI. However, studies that attempted to determine the diagnostic value of DITI for CTS did not yield dependable results. Several studies investigated the diagnostic significance of DITI by comparing affected and unaffected hands of patients with unilateral CTS; however, no consistent conclusions were obtained^11, 19, 49^. Studies comparing the DITI findings of the CTS group and healthy control group also showed inconsistent results regarding the diagnostic value of DITI^43, 50, 51^. Such inconsistencies in previous studies may be primarily attributed to the lack of consideration of disease progression and the small number of hands in the CTS groups used for the analysis. We were able to identify significant changes in DITI findings by dividing CTS into subgroups according to its severity and duration, which is a strength of our research.

As shown in our correlation analysis, it is well-established that changes in small fiber function are not strongly correlated with pain^52^. The subjective pain score is influenced by sensory neural deficits. Although some antidromic C-fibers are associated with sensory symptoms, myelinated Aδ-fibers are involved in pain as the main afferent nociceptor fibers^52, 53^. In the same context, US findings reflect inflammatory changes of the median nerve and carpal tunnel; however, such changes are not directly proportional to the injury of thin-unmyelinated fibers^54^.

Our study had several limitations. Because of the retrospective cross-sectional design of the study, follow-up data regarding disease progression were not obtained. A comparison of DITI results of the same patient over time could further support our findings. Additionally, patients with similar clinical manifestations of CTS and normal findings with electrodiagnosis were excluded from this study. Therefore, direct proof of whether DITI could overcome the aforementioned limitations of electrodiagnosis could not be obtained during this study. Because of the strict inclusion process, we excluded almost half of the hands from the initial dataset. Therefore, for individuals with any complicating factors that could influence DITI, electrodiagnosis, or the subjective pain scale rating, our findings might not be applicable. In terms of clinical information, our study only presented subjective pain scale scores and symptom durations. Because this was a retrospective study, various clinical manifestations, such as the flick sign, hyperalgesia, altered two-point discrimination, night pain, and thenar weakness/atrophy, could not be obtained consistently because the evaluations of clinical symptoms for each patient were not standardized; therefore, some clinical symptoms could have been missed.

In conclusion, DITI findings vary with CTS duration and severity. The temperature of the median nerve-innervated area was higher than that of the ulnar nerve-innervated area, and the difference tended to decrease as the duration and severity increased. Thermal anisometry tended to increase as the duration and severity increased. When interpreting DITI results, it is necessary to consider the symptom duration and disease severity of CTS. Finally, the results of this study using DITI findings elucidated the physiological changes in the unmyelinated autonomic nerve with CTS. Therefore, DITI can have a complementary role when attempting to understand and evaluate CTS, and it can help overcome the shortcomings of electrodiagnosis and US examinations.

## Supplementary Information

Supplementary Information 1.

Supplementary Information 2.

## Data Availability

The dataset including all variables analyzed during this study is available in the online supplementary content.

## References

[1] de Krom MC (1992). Carpal tunnel syndrome: Prevalence in the general population. J. Clin. Epidemiol..

[2] Roh YH, Kim S, Gong HS, Baek GH (2018). Influence of centrally mediated symptoms on functional outcomes after carpal tunnel release. Sci. Rep..

[3] Sonoo M, Menkes DL, Bland JDP, Burke D (2018). Nerve conduction studies and EMG in carpal tunnel syndrome: Do they add value?. Clin. Neurophysiol. Pract..

[4] Rosario NB, De Jesus O (2021). Electrodiagnostic Evaluation of Carpal Tunnel Syndrome.

[5] Moschovos C (2019). The diagnostic accuracy of high-resolution ultrasound in screening for carpal tunnel syndrome and grading its severity is moderated by age. Clin. Neurophysiol..

[6] Csillik A, Bereczki D, Bora L, Aranyi Z (2016). The significance of ultrasonographic carpal tunnel outlet measurements in the diagnosis of carpal tunnel syndrome. Clin. Neurophysiol..

[7] Babaei-Ghazani A (2020). Ultrasound-guided corticosteroid injection in carpal tunnel syndrome: Comparison between radial and ulnar approaches. J. Pain Res..

[8] Maxel X, Bodnar JL, Stubbe L (2014). Detection of carpal tunnel syndrome by infrared thermography. Mech. Ind..

[9] Ming Z, Zaproudina N, Siivola J, Nousiainen U, Pietikainen S (2005). Sympathetic pathology evidenced by hand thermal anomalies in carpal tunnel syndrome. Pathophysiology.

[10] Tattersall GJ (2016). Infrared thermography: A non-invasive window into thermal physiology. Comp. Biochem. Physiol. A..

[11] Nahm FS (2013). Infrared thermography in pain medicine. Korean J. Pain.

[12] Lawson R (1956). Implications of surface temperatures in the diagnosis of breast cancer. Can. Med. Assoc. J..

[13] Choi E, Lee PB, Nahm FS (2013). Interexaminer reliability of infrared thermography for the diagnosis of complex regional pain syndrome. Skin Res. Technol..

[14] Denoble AE, Hall N, Pieper CF, Kraus VB (2010). Patellar skin surface temperature by thermography reflects knee osteoarthritis severity. Clin. Med. Insights Arthritis Musculoskelet. Disord..

[15] Haddad DS, Brioschi ML, Arita ES (2012). Thermographic and clinical correlation of myofascial trigger points in the masticatory muscles. Dentomaxillofac. Radiol..

[16] Elie B, Guiheneuc P (1990). Sympathetic skin response: Normal results in different experimental conditions. Electroencephalogr. Clin. Neurophysiol..

[17] Park ES, Park CI, Jung KI, Chun S (1994). Comparison of sympathetic skin response and digital infrared thermographic imaging in peripheral neuropathy. Yonsei. Med. J..

[18] Santiago S, Ferrer T, Espinosa ML (2000). Neurophysiological studies of thin myelinated (A delta) and unmyelinated (C) fibers: Application to peripheral neuropathies. Neurophysiol. Clin..

[19] Tchou S, Costich JF, Burgess RC, Wexler CE (1992). Thermographic observations in unilateral carpal tunnel syndrome: Report of 61 cases. J. Hand. Surg. Am..

[20] Jesensek Papez B, Palfy M, Mertik M, Turk Z (2009). Infrared thermography based on artificial intelligence as a screening method for carpal tunnel syndrome diagnosis. J. Int. Med. Res..

[21] Baselgia LT, Bennett DL, Silbiger RM, Schmid AB (2017). Negative neurodynamic tests do not exclude neural dysfunction in patients with entrapment neuropathies. Arch. Phys. Med. Rehabil..

[22] Dumitru D, Amato AA, Zwarts MJ (2002). Electrodiagnostic Medicine.

[23] Stevens JC (1997). AAEM minimonograph #26: The electrodiagnosis of carpal tunnel syndrome. American Association of Electrodiagnostic Medicine. Muscle Nerve.

[24] Park D (2021). Electrodiagnostic, sonographic, and clinical features of carpal tunnel syndrome with bifid median nerve. J. Pain Res..

[25] Kim HS, Joo SH, Cho HK, Kim YW (2013). Comparison of proximal and distal cross-sectional areas of the median nerve, carpal tunnel, and nerve/tunnel index in subjects with carpal tunnel syndrome. Arch. Phys. Med. Rehabil..

[26] Campero M, Verdugo RJ, Ochoa JL (1993). Vasomotor innervation of the skin of the hand: A contribution to the study of human anatomy. J. Anat..

[27] Allen RJ, Jefferson EM, Koshi LR (2004). Vasomotor innervation fields of peripheral nerves supplying the hand. J. Neurol. Phys. Ther..

[28] Brelsford KL, Uematsu S (1985). Thermographic presentation of cutaneous sensory and vasomotor activity in the injured peripheral nerve. J. Neurosurg..

[29] Keir PJ, Rempel DM (2005). Pathomechanics of peripheral nerve loading: Evidence in carpal tunnel syndrome. J. Hand Ther..

[30] Kim MS (2012). Skin temperature changes following sciatic nerve injury in rats. J. Neurotrauma.

[31] Sener HO, Tascilar NF, Balaban H, Selcuki D (2000). Sympathetic skin response in carpal tunnel syndrome. Clin. Neurophysiol..

[32] Bennett GJ, Ochoa JL (1991). Thermographic observations on rats with experimental neuropathic pain. Pain.

[33] Sunderland S (1978). Nerves and Nerve Injuries.

[34] Zyluk A, Kosowiec L (2008). Regional sympathetic disturbances in carpal tunnel syndrome: A review. Chir. Narzadow. Ruchu. Ortop. Pol..

[35] Sacharuk VZ (2011). Thermographic evaluation of hind paw skin temperature and functional recovery of locomotion after sciatic nerve crush in rats. Clinics.

[36] Pulst SM, Haller P (1981). Thermographic assessment of impaired sympathetic function in peripheral nerve injuries. J. Neurol..

[37] Wagatsuma A, Osawa T (2006). Time course of changes in angiogenesis-related factors in denervated muscle. Acta Physiol..

[38] van Marken Lichtenbelt WD, Schrauwen P (2011). Implications of nonshivering thermogenesis for energy balance regulation in humans. Am. J. Physiol. Regul. Integr. Comp. Physiol..

[39] Dowdy PA, Richards RS, McFarlane RM (1994). The palmar cutaneous branch of the median nerve and the palmaris longus tendon: A cadaveric study. J. Hand. Surg. Am..

[40] Sun PC (2013). Microcirculatory vasomotor changes are associated with severity of peripheral neuropathy in patients with type 2 diabetes. Diab. Vasc. Dis. Res..

[41] Ming Z, Siivola J, Pietikainen S, Narhi M, Hanninen O (2007). Postoperative relieve of abnormal vasoregulation in carpal tunnel syndrome. Clin. Neurol. Neurosurg..

[42] Baic A (2017). Can we use thermal imaging to evaluate the effects of carpal tunnel syndrome surgical decompression?. Medicine.

[43] Hong YP, Ryu KS, Cho BM, Oh SM, Park SH (2006). Evaluation of thermography in the diagnosis of carpal tunnel syndrome: Comparative study between patient and control groups. J. Korean Neurosurg. Soc..

[44] Doughty CT, Bowley MP (2019). Entrapment neuropathies of the upper extremity. Med. Clin. N. Am..

[45] Schmid AB, Bland JD, Bhat MA, Bennett DL (2014). The relationship of nerve fibre pathology to sensory function in entrapment neuropathy. Brain.

[46] Verghese J, Galanopoulou AS, Herskovitz S (2000). Autonomic dysfunction in idiopathic carpal tunnel syndrome. Muscle Nerve.

[47] Lang E, Claus D, Neundorfer B, Handwerker HO (1995). Parameters of thick and thin nerve-fiber functions as predictors of pain in carpal tunnel syndrome. Pain.

[48] Schmid AB, Coppieters MW, Ruitenberg MJ, McLachlan EM (2013). Local and remote immune-mediated inflammation after mild peripheral nerve compression in rats. J. Neuropathol. Exp. Neurol..

[49] Park J, Lee JW, Lee SE, Kim BH, Park D (2019). Diagnostic usefulness of digital infrared thermal image in carpal tunnel syndrome. Clin. Pain.

[50] Meyers S, Cros D, Sherry B, Vermeire P (1989). Liquid crystal thermography: Quantitative studies of abnormalities in carpal tunnel syndrome. Neurology.

[51] Madarász L, Živčák J (2013). Aspects of Computational Intelligence: Theory and Applications: Revised and Selected Papers of the 15th IEEE International Conference on Intelligent Engineering Systems 2011, INES 2011.

[52] Tamburin S (2011). Median nerve small- and large-fiber damage in carpal tunnel syndrome: A quantitative sensory testing study. J Pain.

[53] Beissner F (2010). Quick discrimination of A(delta) and C fiber mediated pain based on three verbal descriptors. PLoS ONE.

[54] Elnady B (2019). Diagnostic potential of ultrasound in carpal tunnel syndrome with different etiologies: Correlation of sonographic median nerve measures with electrodiagnostic severity. BMC Musculoskelet. Disord..

