# Supplementary material for: Application of digital infrared thermography for carpal tunnel syndrome evaluation
Source: Sci Rep. 2021 Nov 9;11:21963. doi: 10.1038/s41598-021-01381-5 (PMC8578627; doi:10.1038/s41598-021-01381-5)
Supplement: Supplementary file 1 — Supplementary Information 1. [file 41598_2021_1381_MOESM1_ESM.pdf]

Application of Digital Infrared Thermography for Carpal Tunnel Syndrome Evaluation

Dougho Park[1], Byung Hee Kim[1], Sang-Eok Lee[1], Dong Young Kim[2], Yoon Sik Eom[2], Jae Man Cho[3], Joong Won Yang[3], Mansu Kim[3], Heum Dai Kwon[3], Jang Woo Lee[4,*]

[1]Department of Rehabilitation Medicine, Pohang Stroke and Spine Hospital, Pohang, Republic of Korea

[2]Department of Orthopedic Surgery, Pohang Stroke and Spine Hospital, Pohang, Republic of Korea

[3]Department of Neurosurgery, Pohang Stroke and Spine Hospital, Pohang, Republic of Korea

[4]Department of Physical Medicine and Rehabilitation, National Health Insurance Service Ilsan Hospital, Goyang, Republic of Korea

Corresponding author: E-mail: medipia@gmail.com

Supplementary Table S1. Digital infrared thermographic imaging values according to the duration and severity groups.

Groups	Region of interest					
	1st digit	2nd digit	3rd digit	5th digit	Thenar	Hypothenar
Duration groups						
D1 (n = 91)	29.84 ± 2.48	29.68 ± 2.50	29.63 ± 2.47	28.81 ± 2.64	31.74 ± 1.87	31.22 ± 1.93
D2 (n = 59)	30.11 ± 2.27	29.93 ± 2.25	29.78 ± 2.25	29.25 ± 2.41	31.88 ± 1.79	31.43 ± 1.91
D3 (n = 57)	29.88 ± 2.04	29.71 ± 2.14	29.51 ± 2.00	29.14 ± 1.22	31.90 ± 1.68	31.59 ± 1.75
D4 (n = 97)	29.70 ± 2.69	29.38 ± 2.81	29.29 ± 2.76	29.07 ± 3.00	31.33 ± 1.95	31.21 ± 2.21
Severity groups						
S1 (n = 73)	29.75 ± 2.59	29.56 ± 2.56	29.44 ± 2.51	28.72 ± 2.73	31.65 ± 1.94	31.25 ± 2.00
S2 (n = 60)	30.60 ± 1.69	30.39 ± 1.74	30.32 ± 1.73	29.82 ± 1.78	32.41 ± 1.23	31.96 ± 1.38
S3 (n = 83)	30.12 ± 2.21	30.00 ± 2.23	29.86 ± 2.14	29.36 ± 2.32	31.90 ± 1.79	31.54 ± 1.88
S4 (n = 88)	29.18 ± 2.74	28.86 ± 2.88	28.76 ± 2.83	28.45 ± 3.15	30.95 ± 1.95	30.76 ± 2.27
Healthy controls (n = 88)	29.81 ± 1.96	29.76 ± 2.02	29.74 ± 2.00	29.66 ± 2.12	31.40 ± 1.75	31.28 ± 1.87

D, symptom duration group; S, severity group.

Supplementary Table S2. *P* values of *post-hoc* analyses between each subgroup.

	ΔM-U digits	Δthenar-hypothenar	ΔM-U territories	Ansiometry
D1 *vs*. D2	0.14	> 0.99	0.16	> 0.99
D1 *vs*. D3	0.006	0.32	0.003	> 0.99
D1 *vs*. D4	< 0.001	< 0.001	< 0.001	0.005
D2 *vs*. D3	> 0.99	> 0.99	> 0.99	> 0.99
D2 *vs*. D4	0.02	0.004	0.001	0.04
D3 *vs*. D4	0.35	0.37	0.13	0.10
S1 *vs*. S2	0.13	> 0.99	0.41	> 0.99
S1 *vs*. S3	0.10	> 0.99	0.17	> 0.99
S1 *vs*. S4	< 0.001	0.22	< 0.001	0.009
S2 *vs*. S3	> 0.99	> 0.99	> 0.99	> 0.99
S2 *vs*. S4	> 0.99	0.07	0.46	< 0.001
S3 *vs*. S4	0.93	0.61	0.45	0.003

D, symptom duration group; S, severity group; ΔM-U digits, difference between median and ulnar digits; Δthenar-hypothenar, difference between thenar and hypothenar area; ΔM-U territories, difference between median and ulnar territories; Anisometry, median nerve-innervated digits anisometry.

Supplementary Table S3. Cross-sectional areas and subjective pain scores according to the duration and severity groups.

Group	CSA	NRS
Duration Groups		
D1 (n = 91)	14.12 ± 3.30	4.10 ± 1.49
D2 (n = 59)	14.40 ± 2.90	4.54 ± 1.62
D3 (n = 57)	15.40 ± 3.78	5.35 ± 1.54
D4 (n = 97)	17.99 ± 5.43	5.33 ± 1.49
Severity Groups		
S1 (n = 73)	13.70 ± 3.00	3.90 ± 1.50
S2 (n = 60)	13.36 ± 2.90	4.28 ± 1.30
S3 (n = 83)	15.70 ± 3.43	5.04 ± 1.58
S4 (n = 88)	18.79 ± 5.21	5.73 ± 1.40

D, symptom duration group; S, severity group; CSA, cross sectional area of the median nerve; NRS, numeric rating scale of pain.
